# A case of successful conversion surgery for locally advanced pancreatic cancer with synchronous triple cancer of the lung and esophagus: a case report

**DOI:** 10.1186/s40792-022-01377-x

**Published:** 2022-01-24

**Authors:** Junya Mita, Tomohiro Iguchi, Norifumi Iseda, Kazuki Takada, Kosuke Hirose, Naoko Miura, Takuya Honboh, Yasunori Emi, Tetsuro Akashi, Seiya Kato, Noriaki Sadanaga, Hiroshi Matsuura

**Affiliations:** 1 Department of Surgery, Saiseikai Fukuoka General Hospital, 1-3-46 Tenjin Chuo-Ku, Fukuoka, 810-0001 Japan; 2 Department of Internal Medicine, Saiseikai Fukuoka General Hospital, 1-3-46 Tenjin Chuo-Ku, Fukuoka, Japan; 3 Division of Pathology, Saiseikai Fukuoka General Hospital, 1-3-46 Tenjin Chuo-Ku, Fukuoka, Japan

**Keywords:** Pancreatic cancer, Esophageal cancer, Lung cancer, Multiple primary cancer, Synchronous triple cancer, Chemotherapy, Conversion surgery

## Abstract

**Background:**

The number of reports of multiple primary cancer (MPC) is increasing because of the advancement in diagnostic imaging technology. However, the treatment strategy for MPCs involving pancreatic cancer is controversial because of the extremely poor prognosis. We herein report a patient with synchronous triple cancer involving the pancreas, esophagus, and lung who underwent conversion surgery after intensive chemotherapy for unresectable locally advanced pancreatic cancer.

**Case presentation:**

A 59-year-old man was admitted to our hospital with epigastric pain, anorexia, and weight loss. Computed tomography and upper gastrointestinal endoscopy revealed that the patient had synchronous triple cancer of the pancreas, esophagus, and lung. While the esophageal and lung cancer were relatively non-progressive, the pancreatic tail cancer had invaded the aorta, celiac axis, and left kidney, and the patient was diagnosed with unresectable locally advanced disease. Because the described lesion could have been the prognostic determinant for this patient, we initiated intensive chemotherapy (gemcitabine plus nab-paclitaxel) for pancreatic cancer. After six courses of chemotherapy, the tumor size shrank remarkably and no invasion to the aorta or celiac axis was observed. No significant changes were observed in the esophageal and lung cancers; endoscopic submucosal dissection could be still a curative treatment for the esophageal cancer. Therefore, we performed curative resection for pancreatic cancer (distal pancreatomy, splenectomy, and left nephrectomy; ypT3N0cM0, ypStage IIA, UICC 8th). Pathologically, complete resection was achieved. The patient then underwent endoscopic submucosal dissection for early esophageal cancer (pT1a[M]-LPM) and video-assisted thoracoscopic right upper lobectomy in combination with right lower partial resection for early lung cancer (pT2aN0M0, pStage IB, UICC 8th). Eight months after pancreatic cancer surgery, the patient is alive and has no sign of recurrence; as a result of the successful treatment, the patient has a good quality of life.

**Conclusions:**

Treatment of MPC is challenging, especially for cases with unresectable tumors. Although synchronous triple cancer can involve unresectable pancreatic cancer, radical resection may be possible after careful assessment of the appropriate treatment strategy and downstaging of unresectable tumors.

## Background

The number of reports of multiple primary cancer (MPC) is currently increasing because of advancements in diagnostic imaging technology. Several studies have reported MPCs involving pancreatic cancer; common extra-pancreatic sites are the colon, breast, prostate, stomach, and thyroid [1–4]. Because the overall 5-year survival rate for pancreatic cancer remains low at ~ 10% [5], the treatment strategy for MPC involving pancreatic cancer has not yet been well investigated.

Here, we present a case of synchronous triple cancer involving the pancreas, esophagus, and lung in which the patient underwent radical resection following successful downstaging for unresectable locally advanced pancreatic cancer by systemic chemotherapy.

## Case presentation

**Fig. 1:**
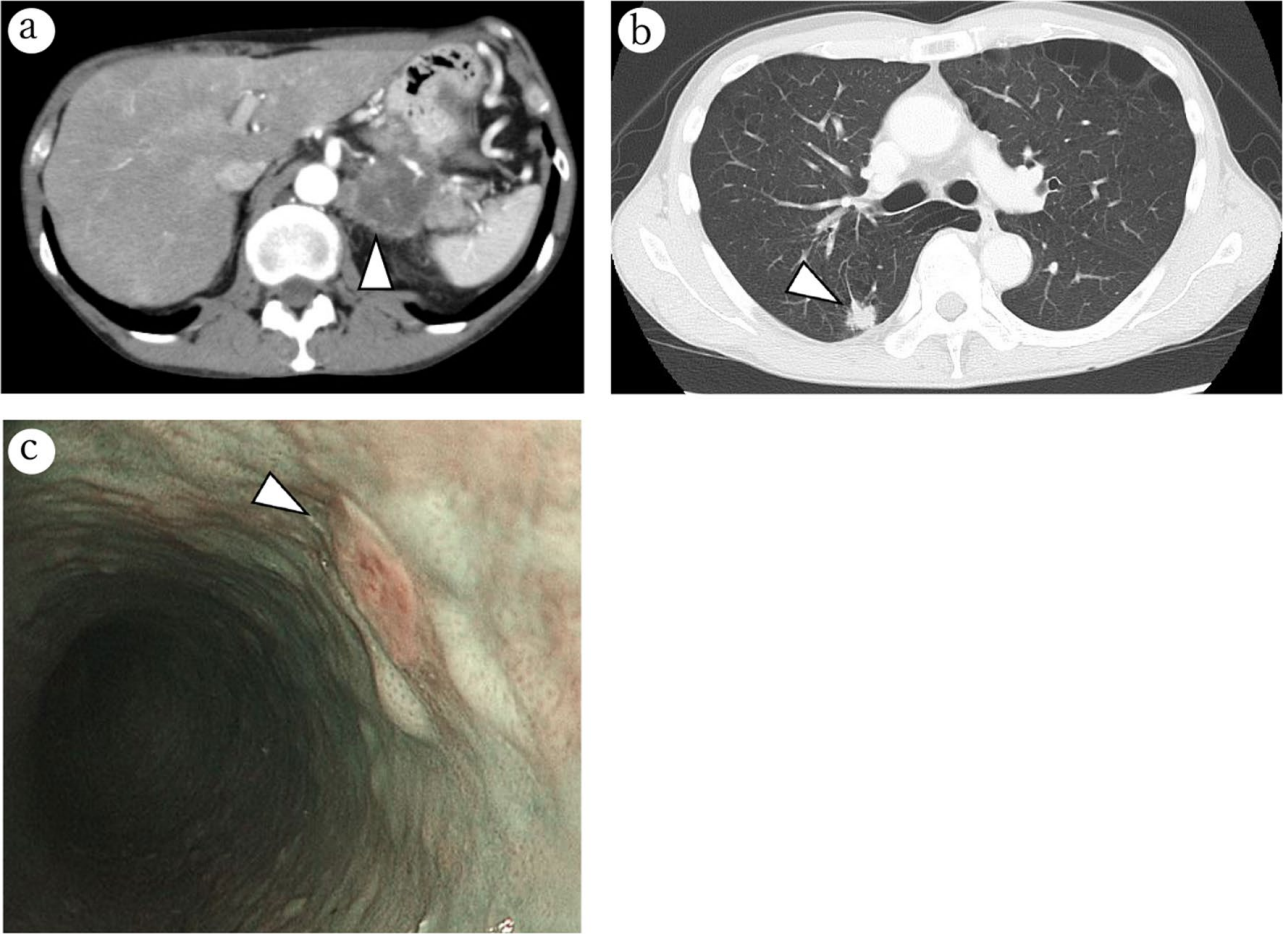
CT and upper gastrointestinal endoscopy findings at presentation. Preoperative CT showed an irregular-shaped mass 55 mm in size at the tail of the pancreas, which was suspected to have caused aortic invasion (arrowhead; **a**). CT showed a tumor of the right S2/6, 17 mm in size (arrowhead; **b**). Upper gastrointestinal endoscopy showed flat early esophageal cancer (0–IIb) located ~ 30 cm from the incisor teeth (arrowhead; **c**)

A 59-year-old man was admitted to our hospital for epigastric pain associated with loss of appetite and weight. Computed tomography (CT) and upper gastrointestinal endoscopy demonstrated that the patient may have had synchronous triple cancer of the pancreas, esophagus, and lung. Tumor markers carcinoembryonic antigen (CEA) and carbohydrate antigen 19-9 (CA19-9) were 6.11 ng/mL and 43.3 U/mL, respectively. A contrast-enhanced CT showed an irregular-shaped mass 55 mm in size at the tail of the pancreas; this mass was suspected to have invaded the aorta, celiac axis, and left kidney; thus, this mass was diagnosed as unresectable locally advanced pancreatic cancer. No lymphadenopathy or distant metastasis was detected by CT (cT4N0M0, cStage III, Union for International Cancer Control (UICC) 8th). Endoscopic ultrasound fine-needle aspiration demonstrated suspected pancreatic adenocarcinoma (class IIIb). An upper gastrointestinal endoscopy was performed to assess malignancy in the upper gastric tract, revealing esophageal squamous cell carcinoma confined to the mucosa, ~ 30 cm from the incisor teeth (cT1aN0M0, cStage IA, UICC 8th). In addition, a 17-mm irregular-shaped lung nodule in the right S2/S6 was detected by CT, which strongly indicated that the patient had primary lung cancer (cT1bN0M0, cStage IA2, UICC 8th; Fig. 1). The clinical diagnosis was synchronous triple cancer involving the pancreas, esophagus, and lung. Although the esophageal and lung cancer were relatively non-progressive and thought to be resectable, the pancreatic cancer was identified as unresectable locally advanced cancer because of the aortic and celiac axis invasion. First, we initiated intensive chemotherapy (gemcitabine plus nab-paclitaxel [GnP]) for pancreatic cancer.

**Fig. 2:**
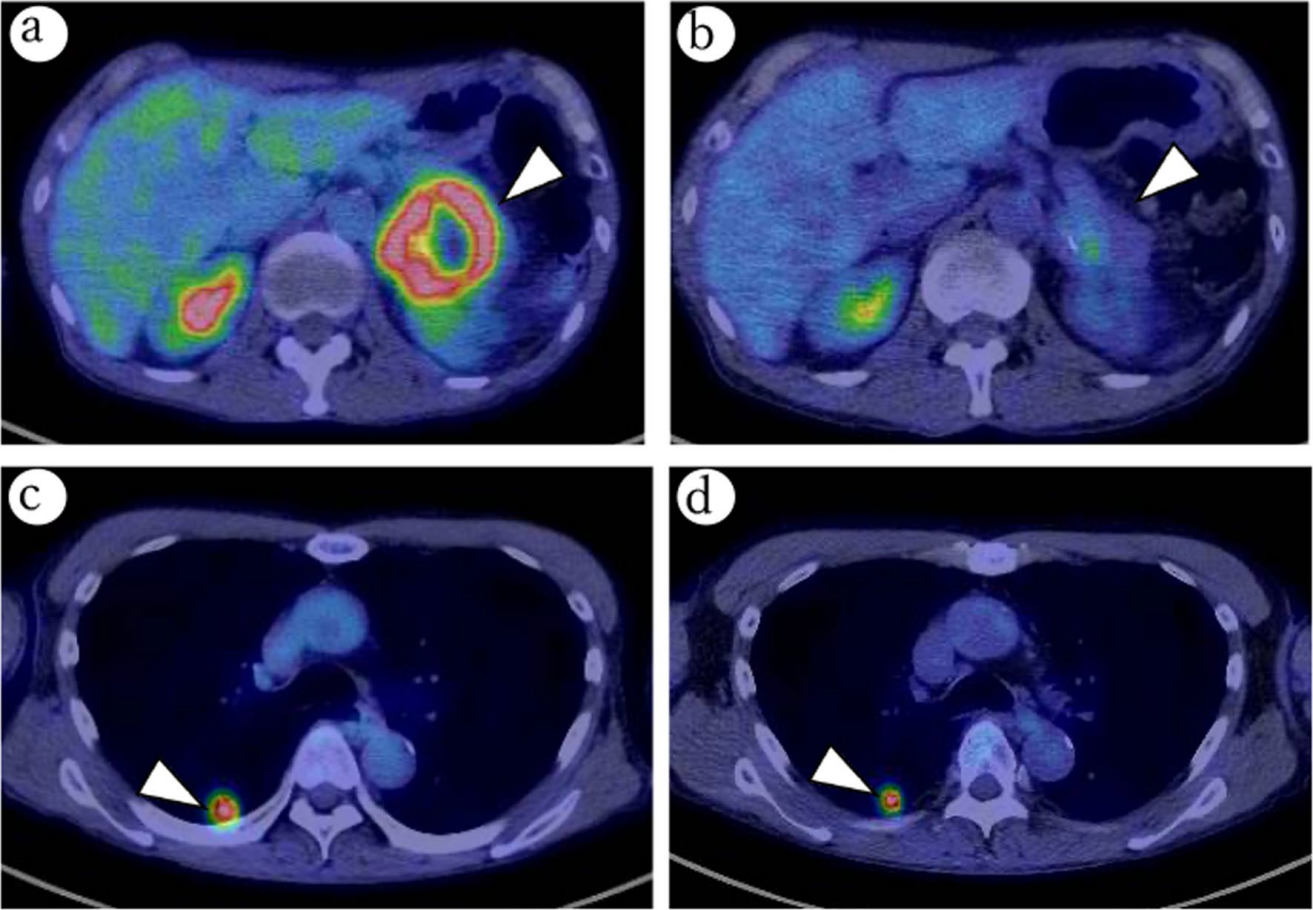
Therapeutic assessments after chemotherapy for pancreatic cancer. Positron emission tomography-CT imaging before (**a**, **c**) and after (**b**, **d**) chemotherapy; [18F]-fluorodeoxyglucose uptake in the pancreatic tumor decreased markedly (maximum standardized uptake value, 9.7–2.5) after chemotherapy (arrowhead; **a**, **b**). No significant changes were observed in the lung cancer (arrowhead; **c**, **d**)

**Fig. 3:**
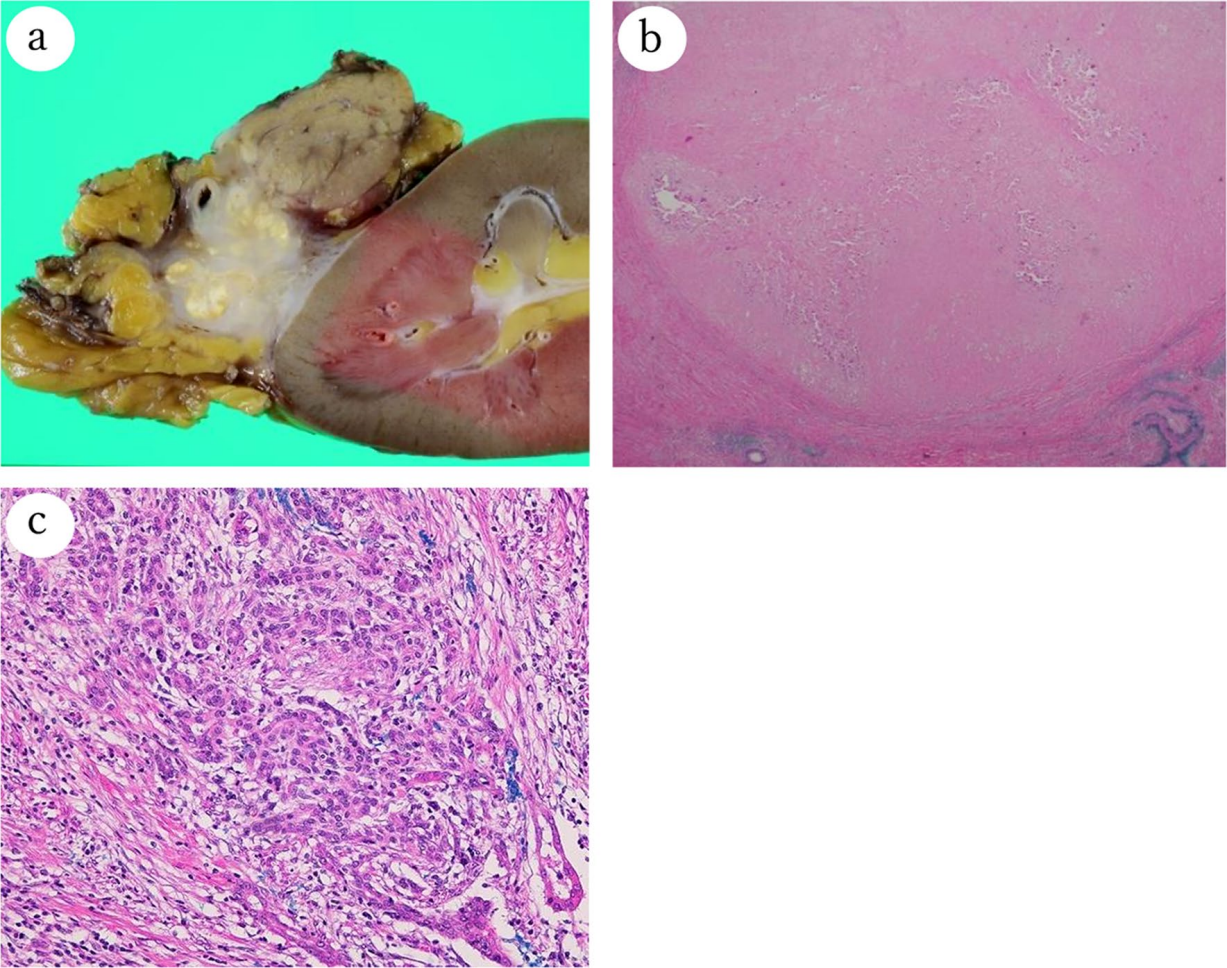
Pathological findings of pancreatic cancer. Macroscopic (**a**) and microscopic (**b**, **c**) examination of the resected specimen; Macroscopically, the ill-demarcated tumor presented in the tail of the pancreas and invaded the left kidney (**a**). The entire tumor showed coagulative tumor necrosis. Viable tumor cells were slightly found in the tumor (hematoxylin–eosin, original magnification ×20; **b**). The residual tumor cells revealed invasive ductal carcinoma (moderately differentiated; hematoxylin–eosin, original magnification ×400; **c**)

After six courses of chemotherapy, CT analysis showed that the pancreatic tumor size had decreased remarkably, and there was no invasion to the aorta or celiac axis. Tumor marker levels had decreased after the treatment; CEA and CA19-9 were 6.08 ng/mL and 10.9 U/mL, respectively. In addition, [18F]-fluorodeoxyglucose uptake, measured by positron emission tomography-CT, in the pancreatic tumor had decreased markedly (maximum standardized uptake value, 9.7–2.5) after chemotherapy. No significant changes were observed in the esophageal and lung cancers, which remained as resectable (Fig. 2); Endoscopic submucosal dissection (ESD) could be still a curative treatment for the esophageal cancer. Therefore, we decided to downstage the locally advanced pancreatic cancer to resectable status. Intraoperative findings showed a solid, movable mass in the tail of the pancreas, separate from the aorta and celiac axis. However, the mass appeared to have invaded the left kidney and the renal vein. The operative procedure was distal pancreatomy, splenectomy, and left nephrectomy. We also performed partial gastrectomy for a 10-mm submucosal tumor (SMT) that was found during the surgery. The operation time was 287 min, and the blood loss was 820 mL. The postoperative course was uneventful, and the patient was discharged on postoperative day 12. Macroscopically, the resected specimen showed an ill-demarcated firm white tumor at the tail of the pancreas, invading the left kidney (Fig. 3a). Tumor necrosis in response to chemotherapy with estimated < 10% residual tumor cells was observed (Miller–Payne grading system, Grade 3; Marked response) (Fig. 3b). The residual tumor cells revealed invasive ductal carcinoma (ypT3N0cM0, ypStage IIA, UICC 8th) (Fig. 3c). SMT of the stomach was histologically confirmed as a gastrointestinal stromal tumor (GIST; pT1aN0M0, pStage IA, UICC 8th; modified Fletcher classification, very low risk). As R0 resection was achieved for the pancreatic cancer and GIST in this patient, we performed curative resection of the other primary cancers. Endoscopic submucosal dissection was performed for the esophageal lesion. The pathological examination indicated well-differentiated squamous cell carcinoma (pT1a[M]-LPM), which was completely resected. Thereafter, video-assisted thoracoscopic right upper lobectomy in combination with right lower partial resection was performed for the lung cancer. The pathological examination showed invasive poorly differentiated adenocarcinoma (pT2aN0M0, pStage IB, UICC 8th). No reduction in the overall tumor cells, which was thought to be no response to the chemotherapy (Miller–Payne grading system, Grade 1; No response). Despite not receiving adjuvant chemotherapy, the patient is alive at time of writing, 8 months after the pancreatic cancer surgery, with no sign of recurrence. As a result of the successful treatment, the patient has a good quality of life.

## Discussion

The incidence of MPC is reported to vary from 2.4 to 18.4% [6, 7] and is defined by Warren and Gates as follows [8]: first, tumors must be histologically different; second, they must exist in different organs; and finally, metastatic lesion of these tumors must be excluded. MPC is divided into two types: synchronous and metachronous. Synchronous MPCs are defined as tumors that are simultaneously diagnosed with additional tumors, or tumors diagnosed within 6 months of the diagnosis of the first tumor [9]. In our case, the MPC involved cancers in three different organs; pancreatic, esophagus, and lung cancer were simultaneously diagnosed, with no evidence of metastasis. The histology of each cancer was distinct from one another and are defined as follows: invasive ductal carcinoma of the pancreas, squamous cell carcinoma of the esophagus, and invasive solid adenocarcinoma of the lung. Though the pancreatic cancer had marked response to the chemotherapy, no response was observed in the lung cancer after the chemotherapy, which strongly suggested a primary lung cancer rather than metastasis of pancreatic cancer. These findings indicate that our case meets the criteria for synchronous triple cancer of the pancreas, esophagus, and lung, which, to our knowledge, has not been previously reported. The GIST was unexpectedly found 7 months after the initial diagnosis of the three cancers during surgery; thus, we categorized the GIST as a metachronous cancer. Considering that GISTs are often slow-growing [10], and that in this case it may have already existed at the time that the other cancers were diagnosed, it could be designated as synchronous quadruple cancer.

Treatment strategies for synchronous triple cancers are not well-defined, because triple cancers are extremely rare and there are only a few reports referring to the frequency and treatment of these cancers. Simultaneous treatment for each cancer considerably increases the treatment volume, adverse effects, and complications; therefore, each cancer often requires separate treatment. Boute et al. reported that the prognosis of MPC could be determined by the prognosis of the most advanced cancer, and that the tumor-node-metastasis stage and histological differentiation may be the major prognostic factors in patients with MPC and single cancers [11]. In our case, we assessed the prognosis of each cancer and determined that the cStage III unresectable pancreatic ductal adenocarcinoma, cStage I esophageal squamous cell carcinoma, and cStage I lung cancer had 5-year overall survival rates of 4.7%, 83%, and 74%, respectively [12–14]. Approximately 10% of patients with pancreatic cancer have one or more extra-pancreatic primary cancers [1]. Hackert et al. reported that no survival difference was noted between pancreatic cancer patients with and without extra-pancreatic primary cancers [2], indicating that the prognosis of MPC that involves pancreatic cancer could be determined by the progression of the pancreatic cancer itself, and that treatment of pancreatic cancer should be given priority over the other primary cancers. Thus, in our case, we decided to initiate chemotherapy for unresectable locally advanced pancreatic cancer, primarily from a prognostic point of view without treating esophageal and lung cancer. The combination of 5-fluorouracil, irinotecan, and oxaliplatin (FOLFIRINOX) and GnP have been used as first-line treatments for locally advanced pancreatic cancer and there was no statistical difference in overall survival between the two regimens [15]. Although FOLFIRINOX including 5-FU and platinum drug may be also effective for the esophageal and the lung cancer, in our case, these lesions were early-stage and resectable without chemotherapy. GnP might be more favorable regimen than FOLFIRINOX with regard to toxicity, especially in Japanese [16]. Thus, we decided to use GnP as first-line treatment.

Of all patients with pancreatic cancer, approximately 70% are not eligible for radical surgery because of locally advanced or metastatic disease at diagnosis; approximately 40% of patients are diagnosed with unresectable locally advanced disease, and 30% are diagnosed with unresectable metastatic disease [17]. Recently, there has been an increasing number of reports on conversion surgery after downstaging of unresectable pancreatic cancer by chemotherapy. According to a randomized study, 35.9% of patients with unresectable locally advanced pancreatic cancer were downstaged and finally underwent successful conversion surgery after GnP therapy [18]. In our case, although the pancreatic cancer was initially suspected to have directly invaded the aorta and celiac axis, which we thought was unresectable, curative resection was achieved after GnP therapy with sequential radical resection of the esophageal and lung cancer.

As pancreatic and esophageal surgeries are invasive, the treatment strategy of MPC involving pancreatic and esophageal cancers is challenging [19]. The type of operation performed for removal of pancreatic cancer based on the tumor location. Especially, pancreaticoduodenectomy for the tumor located in the pancreas head is a highly invasive surgical procedure and esophageal reconstruction could be more complicated [20]. Considering postoperative complications, esophageal reconstruction and abdominal adhesion, it remains controversial whether simultaneous surgery or two-stage surgery is the better management [21]. In our case, as we confirmed the esophageal lesion was endoscopically resectable before the conversion surgery for the pancreatic tail cancer, radical surgery was performed. If esophageal lesion was endoscopically unresectable, the timing and indication for conversion surgery should be considered carefully, especially for pancreatic head cancer.

## Conclusions

Here, we reported a case of a synchronous triple cancer that was radically resected after downstaging of unresectable pancreatic cancer by chemotherapy. Although synchronous triple cancer can involve unresectable lesions, radical resection may be possible once we have established the appropriate treatment strategy for each cancer.

## Data Availability

The authors declare that all the data in this article are available within the article.

## Abbreviations

MPC: Multiple primary cancers
CT: Computed tomography
CEA: Carcinoembryonic antigen
CA19-9: Carbohydrate antigen 19-9
UICC: Union for International Cancer Control
ESD: Endoscopic submucosal dissection
SMT: Submucosal tumor
GnP: Gemcitabine plus nab-paclitaxel
GIST: Gastrointestinal stromal tumor
CK: Cytokeratin
FOLFIRINOX: The combination of 5-fluorouracil, irinotecan, and oxaliplatin
5-FU: Fluorouracil

## References

[1] Riall TS, Stager VM, Nealon WH, Townsend CM, Kuo YF, Goodwin JS, Freeman JL (2007). Incidence of additional primary cancers in patients with invasive intraductal papillary mucinous neoplasms and sporadic pancreatic adenocarcinomas. J Am Coll Surg.

[2] Hackert T, Tjaden C, Müller S, Hinz U, Hartwig W, Strobel O, Fritz S (2012). Extrapancreatic malignancies in patients with pancreatic cancer: epidemiology and clinical consequences. Pancreas.

[3] Lubezky N, Ben-Haim M, Lahat G, Marmor S, Solar I, Brazowski E, Nackache R (2012). Intraductal papillary mucinous neoplasm of the pancreas: associated cancers, family history, genetic predisposition?. Surgery.

[4] Nanashima A, Kondo H, Nakashima M, Abo T, Arai J, Ishii M, Hidaka S (2015). Clinicopathological characteristics of multiple primary cancers in hepatobiliary and pancreas malignancies. Anticancer Res.

[5] Mizrahi JD, Surana R, Valle JW, Shroff RT (2020). Pancreatic cancer. Lancet.

[6] Weir HK, Johnson CJ, Thompson TD (2013). The effect of multiple primary rules on population-based cancer survival. Cancer Causes Control.

[7] Buiatti E, Crocetti E, Acciai S, Gafà L, Falcini F, Milandri C, La Rosa M (1997). Incidence of second primary cancers in three Italian population-based cancer registries. Eur J Cancer.

[8] Warren S (1932). Multiple primary malignant tumors. A survey of the literature and a statistical study. Am J Cancer.

[9] Moertel CG, Bargen JA, Dockerty MB (1958). Multiple carcinomas of the large intestine: a review of the literature and a study of 261 cases. Gastroenterology.

[10] Rubin BP, Heinrich MC, Corless CL (2007). Gastrointestinal stromal tumour. Lancet.

[11] Boute P, Page C, Biet A, Cuvelier P, Strunski V, Chevalier D (2014). Epidemiology, prognosis and treatment of simultaneous squamous cell carcinomas of the oral cavity and hypopharynx. Eur Ann Otorhinolaryngol Head Neck Dis.

[12] Egawa S, Toma H, Ohigashi H, Okusaka T, Nakao A, Hatori T, Maguchi H (2012). Japan Pancreatic Cancer Registry; 30th year Anniversary: Japan Pancreas Society. Pancreas.

[13] Watanabe M (2018). Recent topics and perspectives on esophageal cancer in Japan. JMA J.

[14] Guerrera F, Errico L, Evangelista A, Filosso PL, Ruffini E, Lisi E, Bora G (2015). Exploring stage I non-small-cell lung cancer: development of a prognostic model predicting 5-year survival after surgical resection†. Eur J Cardiothorac Surg.

[15] Arima S, Kawahira M, Shimokawa M, Ido A, Koga F, Ueda Y, Nakazawa J (2021). Gemcitabine plus nab-paclitaxel versus FOLFIRINOX in locally advanced, unresectable pancreatic cancer: a multicenter observational study (NAPOLEON Study). Pancreas.

[16] Muranaka T, Kuwatani M, Komatsu Y, Sawada K, Nakatsumi H, Kawamoto Y, Yuki S, Kubota Y, Kubo K, Kawahata S, Kawakubo K, Kawakami H, Sakamoto N (2017). Comparison of efficacy and toxicity of FOLFIRINOX and gemcitabine with nab-paclitaxel in unresectable pancreatic cancer. J Gastrointest Oncol.

[17] Yamada S, Fujii T, Yokoyama Y, Kawashima H, Maeda O, Suzuki K, Okada T (2018). Phase I study of chemoradiotherapy using gemcitabine plus nab-paclitaxel for unresectable locally advanced pancreatic cancer. Cancer Chemother Pharmacol.

[18] Kunzmann V, Siveke JT, Algül H, Goekkurt E, Siegler G, Martens U, Waldschmidt D (2021). Nab-paclitaxel plus gemcitabine versus nab-paclitaxel plus gemcitabine followed by FOLFIRINOX induction chemotherapy in locally advanced pancreatic cancer (NEOLAP-AIO-PAK-0113): a multicentre, randomised, phase 2 trial. Lancet Gastroenterol Hepatol.

[19] Fukaya M, Abe T, Yokoyama Y, Itatsu K, Nagino M (2014). Two-stage operation for synchronous triple primary cancer of the esophagus, stomach, and ampulla of Vater: report of a case. Surg Today.

[20] Asai S, Fukaya M, Fujieda H, Igami T, Tsunoda N, Sakatoku Y, Kamei Y (2019). Esophageal reconstruction using a pedicled jejunum following esophagectomy for metastatic esophageal stricture from breast cancer in a patient with previous pancreatoduodenectomy. Nagoya J Med Sci.

[21] Izumi H, Yoshii H, Abe R, Yamamoto S, Mukai M, Nomura E, Sugiyama T (2019). Pancreaticoduodenectomy following surgery for esophageal cancer with gastric tube reconstruction: a case report and literature review. Surg Case Rep.

